# Neuromedin U Neurons in the Edinger–Westphal Nucleus Respond to Alcohol Without Interfering with the Urocortin 1 Response

**DOI:** 10.1007/s11064-024-04238-1

**Published:** 2024-09-12

**Authors:** Mireia Medrano, Wissal Allaoui, Ra’fat Ehab Salim Haddad, Leila Makrini-Maleville, Emmanuel Valjent, Ilse Smolders, Viktória Kormos, Balázs Gaszner, Dimitri De Bundel

**Affiliations:** 1 Center for Neurosciences, Department of Pharmaceutical Chemistry, Drug Analysis and Drug Information, Research Group Experimental Pharmacology, Vrije Universiteit Brussel, Laarbeeklaan 103, 1090 Brussels, Belgium; 2 Medical School, Research Group for Mood Disorders, Department of Anatomy and Centre for Neuroscience, University of Pécs, Szigeti út 12, 7624 Pécs, Hungary; 3 IGF, Université de Montpellier, CNRS, Inserm, Montpellier, France; 4 Medical School, Department of Pharmacology and Pharmacotherapy, University of Pécs, Szigeti út 12, 7624 Pécs, Hungary

**Keywords:** Neuromedin U, Urocortin 1, Alcohol, Edinger–Westphal nucleus, Knock-in mouse model

## Abstract

The Edinger–Westphal nucleus (EW) is a midbrain nucleus composed of a preganglionic, cholinergic subpopulation and a densely clustered peptidergic subpopulation (EWcp). The EWcp is one of the few brain regions that show consistent induction of FOS following voluntary alcohol intake. Previous results in rodents point to urocortin 1 (UCN1) as one of the peptides most involved in the control of ethanol intake and preference. Notably, the functions described for UCN1, such as reward processing, stress coping or the regulation of feeding behavior are similar to those described for the neuropeptide neuromedin U (NMU). Interestingly, NMU has been recently associated with the modulation of alcohol-related behaviors. However, little is known about the expression and functionality of NMU neurons in alcohol-responsive areas. In this study, we used the recently developed Nmu-Cre knock-in mouse model to examine the expression of NMU in the subaqueductal paramedian zone comprising the EWcp. We delved into the characterization and co-expression of NMU with other markers already described in the EWcp. Moreover, using FOS as a marker of neuronal activity, we tested whether NMU neurons were sensitive to acute alcohol administration. Overall, we provided novel insights on NMU expression and functionality in the EW region. We showed the presence of NMU within a subpopulation of UCN1 neurons in the EWcp and demonstrated that this partial co-expression does not interfere with the responsivity of UCN1-containing cells to alcohol. Moreover, we proposed that the UCN1 content in these neurons may be influenced by sex.

**Supplementary Information:**

The online version contains supplementary material available at 10.1007/s11064-024-04238-1.

## Introduction

The Edinger–Westphal (EW) nucleus is a compact, midbrain structure that is traditionally perceived as a brain region involved in oculomotor adaptation [1, 2]. It is now recognized that the EW is composed of two major subpopulations of neurons: the preganglionic EW (EWpg) is a parasympathetic cholinergic brain structure, part of the oculomotor nuclear complex, and the EW comprising peptidergic centrally projecting neurons (EWcp) is involved in stress coping, reward processing and consumptive functions [3]. The EWcp is sensitive to several addictive drugs, as measured by increased FOS protein expression after exposure to ethanol [4–8], morphine [9], heroin [10] and psychostimulants such as cocaine and methamphetamine [6] in both mice and rats. The EWcp is one of the few brain regions that consistently showed induction of FOS across rodent species upon voluntary alcohol intake [5–8]. Moreover, FOS in the EWcp shows a positive correlation with the amount of alcohol consumed [11–13]. In this line, direct pharmacogenetic manipulation of EWcp neurons was shown to influence ethanol consumption in mice [14, 15]. Together, these findings indicate a key role of the EWcp in alcohol consumption [5, 8, 15–18].

Neurons in the EWcp express various neuropeptides including cocaine- and amphetamine-regulated transcript (CART), substance P (SP), cholecystokinin (CCK), pituitary adenylate cyclase-activating polypeptide (PACAP), urocortin 1 (UCN1) and neuromedin U (NMU) [19–21]. Some of these neuropeptides has been shown to be sensitive to alcohol, such as CART and SP [22–25]. However, UCN1 received particular attention as one of the main peptides involved in the control of ethanol preference and intake in rodents [5, 8, 11, 15, 17, 18, 26]. UCN1 is a member of the corticotropin-releasing hormone (CRH) family, primarily expressed in the EWcp [27, 28]. Alcohol-induced expression of FOS in the EWcp co-localized extensively with UCN1 [5, 29] and knockdown of UCN1 in the EWcp reduced escalation of ethanol intake in mice [11]. Notably, high alcohol preference in both mice and rats has been associated with increased UCN1 levels [16, 29–32]. Interestingly, the functions of UCN1, such as reward processing, stress coping, energy homeostasis and regulation of feeding behavior, are comparable to those described for NMU, which also shares some UCN1 expression sites and interacts with the CRH system in numerous ways [20, 33–35].

Recently, NMU has been shown to influence the reinforcing effects of drugs and modulate reward-related behaviors in rodents and humans [36, 37]. Notably, a role in alcohol-related behaviors has been suggested [38–41]. In contrast to UCN1, NMU administration was shown to reduce ethanol-induced place preference in mice and ethanol intake in rats [41, 42]. However, it remains unknown whether NMU neurons of the EWcp regulate ethanol preference and intake. Intriguingly, pharmacogenetic activation of non-UCN1-containing neurons of the EWcp suppressed ethanol intake in mice [15]. We therefore wondered whether NMU is expressed by a distinct population of EWcp peptidergic neurons that may oppose the activation of UCN1 neurons.

In the present study, we examined the expression of NMU in the subaqueductal paramedian zone, the neuroanatomical region comprising the EWcp, and we addressed the question whether NMU neurons in this area are sensitive to alcohol exposure by means of FOS protein expression. Moreover, we studied the co-expression of NMU with UCN1 in the EWcp, and whether NMU interferes with the responsivity of UCN1-containing cells to alcohol. Altogether, we provide new information on NMU expression in the EW region and novel insights into the functionality of NMU and UCN1 in response to alcohol.

## Materials and Methods

### Animals

The B6.NmuCre-IRES-Nmu or Nmu-Cre knock-in mouse line was developed in collaboration with genOway (France) on a C57BL/6 genetic background as described before [43]. Nmu-Cre heterozygous mice were backcrossed to C57BL/6 J mice (Janvier, France) to maintain the breeding colony. B6.Cg-*Gt*(*ROSA*)*26Sor*^*tm6*(*CAG−ZsGreen1*)*Hze*^/J, also known as Ai6 or Ai6(RCL-ZsGreen) [44] mice (stock #007906, The Jackson Laboratory, USA; RRID IMSR_JAX:007906) were used as Cre reporter line. Male and female Nmu-Cre heterozygous mice were crossed with homozygous Ai6 reporter mice to obtain Nmu-Cre:ZsGreen1 offspring that drive expression of the green fluorescent protein ZsGreen1 in a Cre-dependent manner. All mice were bred in-house at the animal facility of the Vrije Universiteit Brussel. All mice were adult (≥ 8 weeks) when set in breeding or at the start of experiments. Mice were group-housed (1290 eurostandard type III cages, Tecniplast, Italy) in a temperature (18–24 °C) and humidity (30–70%) regulated environment with a 12/12h light/dark cycle (onset dark cycle: 6 p.m.) and had free access to food pellets (A03, SAFE, France) and water. Cages were minimally enriched with shelters, wooden gnawing blocks and nesting material. Nmu-Cre mice undergoing stereotaxic surgery were then housed in individual cages (1264C Eurostandard type II, Tecniplast, Italy) for the remainder of the experiment. All mice used in the present study were sacrificed between 3 and 5 p.m. All experiments were conducted by certified and experienced researchers and were approved by the Ethical Committee for Animal Experiments of the Faculty of Medicine and Pharmacy of the Vrije Universiteit Brussel. All experiments were performed according to the European Community Council directive (2010/63/EU) and the Belgium Royal Decree (29/05/2013) and complied with the ARRIVE guidelines [45]. All efforts were made to reduce stress and suffering of the animals to a minimum.

### Genotyping

Offspring of the cross between Nmu-Cre knock-in and C57BL/6J mice or between Nmu-Cre knock-in and Ai6 reporter mice were genotyped by PCR using REDExtract-N-Amp™ Tissue PCR Kit (#R4775; Sigma-Aldrich, Germany). Primers (Eurogentec, Belgium) were designed to identify the presence or absence of the knock-in allele (5′-GTGACAGGAGAGGAGATGCGGTTGC-3′ [forward primer] and 5′-AGCAAGAGGAGGCGCACAGGA-3′ [reverse primer] to detect the wild-type allele [178 bp]; and 5′-GTGACAGGAGAGGAGATGCGGTTGC-3′ [forward primer] and 5′-ACCTTGGCCTCCCAAATTGCTG-3′ [reverse primer] to detect the neo-excised knock-in allele [325 bp]. The PCR amplification reaction was carried out under the following conditions: 94 °C for 2 min, 30 cycles of 94 °C for 30 s, 65 °C for 30 s and 68 °C for 5 min, and a final extension step at 68 °C for 8 min. PCR reaction products were separated on a 2% agarose gel electrophoresis and visualized with GelRed (nucleic acid gel stain; #41003, Biotium, USA).

### Viral transduction

Viral transduction of NMU-expressing cells was achieved by intracerebral injection of the double-floxed adeno-associated viral (AAV) vector AAV5-EF1a-DIO-RFP (Vector Biosystem, USA), as previously described [43]. Briefly, mice were deeply anesthetized with 2–3% isoflurane (1000 mg/g, Vetflurane Neurology, Virbac, Belgium) and mounted on a stereotaxic frame. Anaesthesia was maintained during the entire duration of the surgery using 1–2% isoflurane in 100% oxygen, delivered via an inhalation cone. Meloxicam (5 mg/kg, Metacam®, 5 mg/mL, Boehringer Ingelheim, Germany) was administered subcutaneously to prevent postoperative pain and inflammation. The AAV vector (titer 1.2 × 10^12^ gc/mL; 500 nL) was unilaterally infused into the target region at specific coordinates relative to Bregma (antero-posterior (AP) − 2.75 mm, medial–lateral (ML) + 0.77 mm and dorso-ventral (DV) − 4.42 mm, 10-degree angle) at a flow rate of 0,15 μL/min using a 10 μL microsyringe (Hamilton Neuros, USA) in a microinjector unit (Model 5001, Kopf®, USA). At the end of the surgical procedure, mice received 1 mL saline (0,9% NaCl, Baxter, Belgium) intraperitoneal (i.p.) and were placed in a recovery box with heating (ThermaCage®, Datesand Group, UK). Once fully awake and responsive mice were returned to their home cage to allow further recovery. Mice were single-housed to prevent damage to the suture and sacrificed for ex vivo experiments after 3 weeks, time needed for proper transduction of the AAV vector and full expression of red fluorescent protein (RFP).

### Tissue Preparation for Histology

Mice were deeply anesthetized by an i.p. overdose of sodium pentobarbital (250 mg/kg Dolethal®, Vetoquinol, France). Transcardiac perfusion was performed immediately after respiratory arrest, using phosphate-buffered saline (PBS, pH 7,4, Sigma-Aldrich, Germany) followed by 4% paraformaldehyde (PFA, VWR International, Belgium) in PBS (pH 7,4) for 5 min at a rate of 10 mL/min. Next, brains were dissected and post-fixed overnight with PFA 4% and then stored in Tris-buffered saline (TBS) solution (50 mM Tris, pH 7,6, Sigma-Aldrich, Germany) at 4 °C. Coronal sections (40 μm) were prepared using a vibratome (Leica VT1000S, Leica Biosystems, Germany) and stored at − 20 °C in an anti-freeze solution (30% glycerol [Merck Millipore, Germany], 30% ethylene glycol [VWR International, USA] and 10% TBS) until further processing. Free-floating sections were selected and rinsed three times with TBS, each for 10 min. Then, sections were pretreated with TBS containing 0.1% Triton-X (TBS-T; Sigma-Aldrich, Germany) for 15 min at room temperature, and incubated with 25 µg/mL of DAPI (4’,6-diamidino-2-phenylindole dihydrochloride; Cell Signaling Technology, USA) for 5 min. Finally, the slices were washed two times with Tris buffer (TB, 50 mM Tris, pH 7.6, Sigma-Aldrich, Germany), mounted on Superfrost slides (Superfrost plus, VWR international, Belgium) and coverslipped using Dako mounting medium (Agilent, USA). Endogenous ZsGreen1 fluorescence and DAPI staining were evaluated using a confocal laser scanning microscope (Zeiss, Axio Observer with LSM 710-6NLO configuration, Zeiss International, Germany) and analyzed using Image J software (NIH, USA; RRID:SCR_003070).

### Immunohistochemistry

Free-floating sections were selected and rinsed three times for 10 min with TBS. Next, sections were pretreated with TBS-T containing 10% normal donkey serum (NDS, Merck Millipore, USA) for 1 h at room temperature under gentle agitation. Next, sections were incubated overnight in primary antibodies (Table 1) at 4 °C. The next day, sections were rinsed three times with TBS-T and incubated with secondary antibodies (Table 2) for 45 min at room temperature and protected from light. DAPI staining and mounting were performed as described [43]. Fluorescent labelling was visualized with a confocal laser scanning microscope (Zeiss, Axio Observer with LSM 710-6NLO configuration, Zeiss International, Germany), and analyzed using Image J software (NIH, USA; RRID:SCR_003070). DAPI staining was used to identify fiber tracts, ventricles and reference regions and estimate the antero-posterior levels relative to Bregma in each slice. ImageJ software (*BigWarp* plugin) was used to superimpose the images with the boundaries defined in the Paxinos and Franklin Atlas (Paxinos & Franklin, 2007). The specificity of all the other primary antibodies used in this study was determined by Western blot analysis (see supplier’s information, Table 1).

**Table 1:** Primary antibodies

Antibody	Class	Host species	Manufacturer (catalogue nº)	Dilution	RRID*
Anti-mCherry	Polyclonal	Goat	LifeSpan BioSciences, USA (LS-C204207)	1:500	AB_2619713
Anti-tryptophan Hydroxylase 2 (TPH2)	Polyclonal	Rabbit	Abcam, UK (ab111828)	1:1000	AB_10862137
Anti-Choline Acetyltransferase (ChAT)	Polyclonal	Rabbit	Millipore, USA (AB143)	1:100	AB_2079760
Anti-tyrosine Hydroxylase (TH)	Polyclonal	Rabbit	Millipore, USA (AB152)	1:1000	AB_390204
Anti- cholecystokinin-8 (CCK8)	Monoclonal	Mouse	Abcam, UK (ab37274)	1:250	AB_726010
Anti-c-Fos (FOS)	Monoclonal	Rabbit	Cell Signaling, USA (2250S)	1:500	AB_310305
Anti-c-Fos (FOS)	Polyclonal	Guinea pig	Synaptic Systems, Germany (226 005)	1:800	AB_2800522
Anti-UCN1	Polyclonal	Rabbit	The Salk Insititute, USA (Prof. Wylie W. Vale)	1:20,000	AB_2315527
Anti-RFP	Monoclonal	Rat	ChromoTek and Proteintech Ltd, Germany (5F8)	1:10,000	AB_2336064

^*^RRID Research Resource Identifier—https://scicrunch.org/resources

**Table 2:** Secondary antibodies

Conjugate	Target	Host species	Catalogue n°	Dilution	RRID^*^
Cyanine Cy^TM^5	Rabbit	Donkey	711-175-152	1:400	AB_2340607
Cyanine Cy^TM^3	Rabbit	Donkey	711-165-152	1:400	AB_2307443
Cyanine Cy^TM^3	Goat	Donkey	705-165-147	1:400	AB_2307351
Cyanine Cy^TM^5	Mouse	Goat	115-175-146	1:400	AB_2338713
Cyanine Cy^TM^3	Rat	Donkey	712-165-150	1:500	AB_2340666
Alexa Fluor 647	Rabbit	Donkey	711-605-152	1:500	AB_2492288
Alexa Fluor 488	Guinea pig	Donkey	706-545-148	1:500	AB_2340472
Alexa Fluor 488	Rabbit	Donkey	711-545-152	1:500	AB_2313584
Alexa Fluor 488	Mouse	Donkey	715-545-150	1:400	AB_2340846
Alexa Fluor 488	Rabbit	Goat	111-545-003	1:400	AB_2338046

All secondary antibodies used were ordered from Jackson ImmunoResearch (USA)

^*^RRID Research Resource Identifier—https://scicrunch.org/resources

The triple labeling for RFP, FOS and UCN1 was performed also on free floating sections. After washes in PBS, antigen retrieval in citrate buffer (pH = 6, 10 min, 90 °C) was performed followed by Triton-X 100 treatment (0.5% in PBS for 60 min, Sigma-Aldrich, Germany). Next, the sections were blocked with NDS (2% in PBS) for 60 min. The primary antibody cocktail containing rat polyclonal anti-RFP antibodies (1:10.000, ChromoTek and Proteintech Ltd, Planegg-Martinsried, Germany, Cat No: 5F8), rabbit polyclonal anti-UCN1 (1:20.000, Prof. Wylie W. Wale, The Salk Institute, La Jolla, CA, USA), and guinea pig polyclonal anti-FOS (1:800, anti c-Fos, Synaptic Systems Cat No: 226 005) in 2% NDS-containing PBS overnight at room temperature. On the second day, sections were rinsed with PBS, and a cocktail of fluorophore-conjugated secondary antibodies (Cyanine (Cy) 3-conjugated donkey anti-rat, Alexa Four (AF) 488-conjugated donkey anti guinea pig and AF 647-conjugated donkey anti-rabbit; for further details see Table 2) were used in 2% NDS for 3 h. Finally, sections were washed with PBS and mounted on gelatin-covered slides, air-dried and covered with glycerol-PBS (1:1) solution.

The intensity of UCN1 immunofluorescence was semi-quantified by measurement of the cytoplasmic signal corrected for the background, yielding the specific signal density (SSD) as published before [47]. The UCN1 SSD was determined in EWcp cells that were positive for RFP and also in those that did not contain this signal.

### RNA In situ Hybridization

#### RNAscope® Protocol

Adult Nmu-Cre mice were sacrificed by cervical dislocation, and brains were removed, placed immediately on dry ice for 5 min and stored at − 80 °C. 14 µm thick sections were obtained at -17 °C with a cryostat. Slices were collected onto Superfrost Plus slides (Thermo Fisher Scientific, USA). Probes for *Nmu* (#446831-C3), *Slc32a1* (#319191-C2), *Slc17a6* (#319179-C1), *Cck* (#402271-C2), *Cartpt* (#432001-C2) and *Tac1* (#410351-C1) were used with the RNAscope® Fluorescent Multiplex labeling kit (#320850; Advanced Cell Diagnostics) according to the manufacturer’s recommendations.

Slides were mounted with ProLong Diamond Antifade mountant (#P36961, Invitrogen, USA). Single-molecule fluorescence of labeled cells were captured using sequential laser scanning confocal microscopy (Leica SP8, Germany) and analyzed using Image J software (NIH, USA; RRID:SCR_003070). DAPI staining was used to identify fiber tracts, ventricles and reference regions and estimate the antero-posterior levels relative to Bregma in each slice.

#### RNAscope® Protocol Combined with Immunofluorescence

Adult Nmu-Cre mice were deeply anesthetized by an i.p. overdose of sodium pentobarbital (250 mg/kg Dolethal®, Vetoquinol, France) and perfused with 4% PFA in 0.1 M Millonig’s phosphate buffer. Dissected brains were postfixed for 72 h at 4 °C, rinsed in 1 × phosphate buffer saline (PBS) and sectioned (30 µm thickness) using a vibrating microtome (VT1000S, Leica Biosystems, Wetzlar, Germany). Sections were stored in 1 × PBS with 0.01% Na-azide (Merck KGaA, Darmstadt, Germany). The pretreatment procedure for RNAscope® was optimized for 30 μm paraformaldehyde-fixed sections, as we previously published [48]. The additional steps of the RNAscope® protocol was performed based on the manufacturer’s instructions for the RNAscope Multiplex Fluorescent Reagent Kit (version 2; ACD). We used a mouse *Nmu* probe (Cat. No.: 446831; ACD), visualized by Cy3 (1:750) and a mouse *Ucn1* probe (Cat. No.: 466261; ACD) which was labelled with fluorescein (FITC). The co-expression of the UCN1 at protein level was also confirmed in sections that were hybridized with *Nmu* probes only. Here, after channel development, slides were subjected to immunofluorescence using polyclonal rabbit anti-UCN1 anti-serum for 24 h at 24 °C. After washes, we used AF 488-conjugated donkey anti-rabbit serum for 3 h. Finally, to visualize the cell nuclei, sections were counterstained with 4′,6-diamidino-2-phenylindole (DAPI; ACD) and covered with ProLong Gold Antifade mounting medium (Thermo Fisher Scientific).

Some randomly selected sections of the EWcp area were also hybridized with triplex positive control probes for the mouse (Cat. No.: 320881; ACD) or with a triplex negative control (Cat. No.: 320871; ACD). After channel development, the positive control probes provided clear fluorescent signal puncta in the low- (DNA-directed RNA polymerase II subunit RPB1 mRNA, *Polr2a*) and mid-copy (Peptidyl–prolyl cis–trans isomerase B mRNA, *Ppib*) channels. Moreover, we saw a confluent fluorescence with the high-copy (Polyubiquitin-C mRNA, *Ubc*) positive control. No fluorescence was recognizable in any channels when a negative control probe, designed to recognize bacterial dihydrodipicolinate reductase (*dabP*) mRNA, was applied (Suppl. Figure 1). The respective positive and negative control probes were used to select the optimal settings for imaging of our RNAscope® labelings, in order to avoid false positive and negative imaging results. The laser beam intensity, the excitation and detection parameters were set in a way that the individual RNAscope® signal puncta appeared as saturated signal dots. Using the same settings, no signal puncta appeared in the images of negative controls. In the view of the low copy expression of the *Nmu* mRNA, and because the RNAscope® technique allows single mRNA molecule detection, in a case, if a particular signal dot was well-recognizable in a cell, it was considered as positive for the examined mRNA. However, in a case, if a particular signal dot was present in all channels, it was considered as a technical artefact or autofluorescence that was not evaluated as positive signal (see also Fig. 8 in [49]).

### Alcohol-Induced Expression of FOS in the EW

Nmu-Cre mice underwent surgery for viral transduction of NMU-expressing cells by intracerebral injection of the double-floxed AAV5-EF1a-DIO-RFP vector as described in 2.3. After surgery, mice were single-housed to prevent damage to the suture. To make experiments comparable, Nmu-Cre:ZsGreen1 were single-housed throughout the experiment. Experiments were conducted in the light phase of the light/dark cycle between 9:00 AM and 2:00 PM. Experimenters were blinded to treatment throughout the study. 1 week prior to the experiment (Nmu-Cre:ZsGreen1) or 2 weeks after surgery (Nmu-Cre) mice were moved and acclimatized to the test room and were habituated to handling and injections for 3 min twice a day starting four to five days prior to drug administration. The day of the experiment, mice received an i.p. injection of vehicle (NaCl 0,9%) or alcohol (ethanol 2,4 g/Kg, 20% v/v in NaCl 0.9% [density 0.97 g/mL]) and immediately returned to their home cages. To evaluate FOS immunoreactivity, mice were transcardially perfused 90 min after drug treatment.

### Statistical Data Analysis

Graphical representations and statistical analyses were performed using GraphPad Prism 8 (GraphPad Software, Inc., USA, RRID SCR_002798) or Statistica (Statsoft Inc. Tulsa, OK, USA, RRID:SCR 014213) software. Data are expressed in columns with dots representing individual values, with the designation of mean and standard deviation. Data were analyzed using two-way or multifactorial analysis of variance (ANOVA) and Tukey’s multiple comparison test. Boxplots representing quartiles and whiskers corresponding to range were also used, showing dots as individual values. Data were analyzed using non-parametric statistics (Mann–Whitney test, unpaired, two-tailed). Nested graphs were used to represent rostral, midlevel and caudal EW counts as technical replicates per mouse. Data were analyzed using nested t tests, two-tailed. Significance threshold was set at alpha = 0.05.

## Results

### NMU-Containing Cells are Expressed in but not Restricted to the Edinger–Westphal Nucleus

**Fig. 1:**
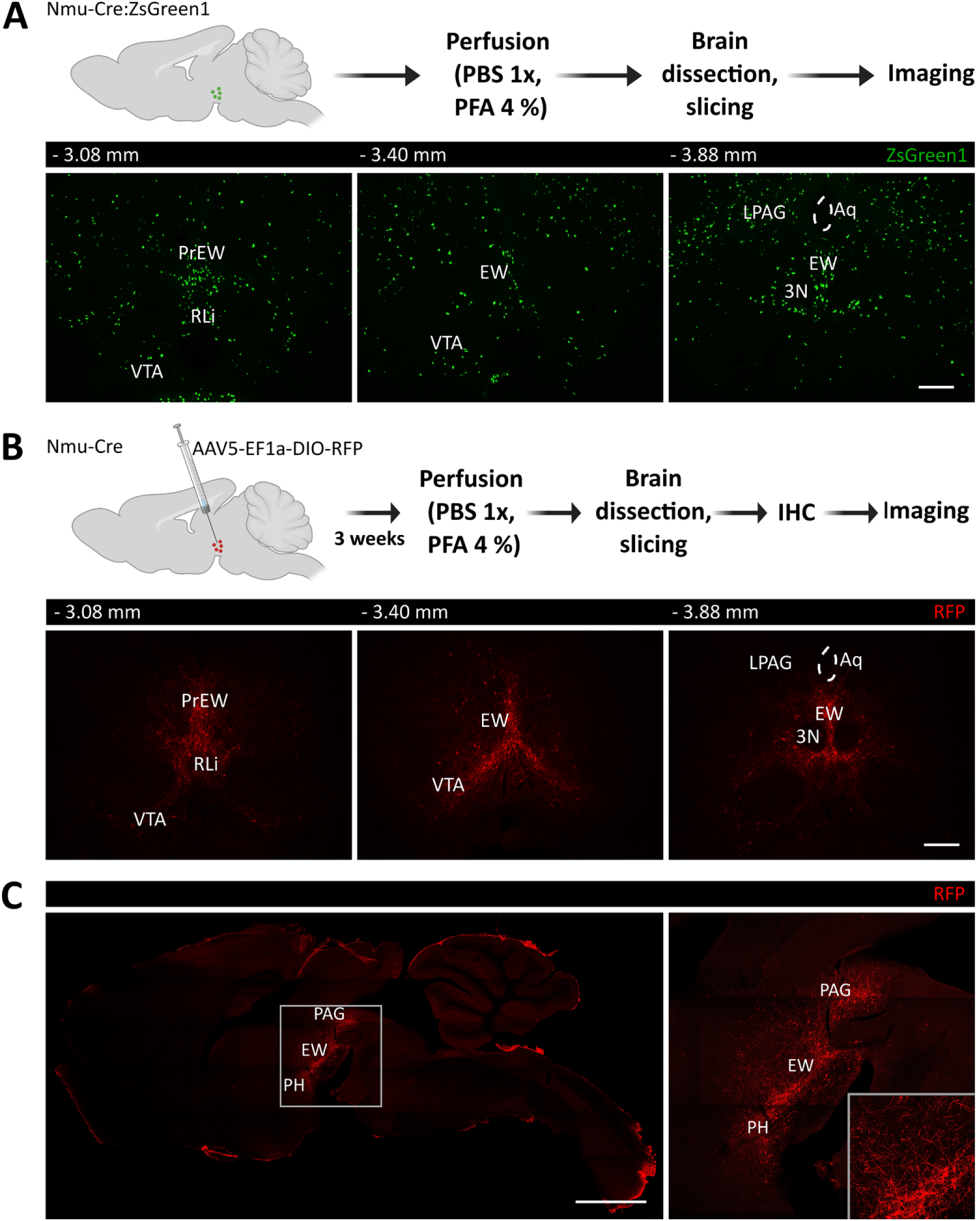
Neuroanatomical distribution of NMU-producing cells in adult mice brain slices. **A** Schematic representation of the protocol followed to prepare samples from Nmu-Cre:ZsGreen1 mice for imaging. Distribution of ZsGreen1-expressing cells in coronal slices showing endogenous ZsGreen1 expression in green. **B** Schematic representation of the intracerebral injection of the viral tracer AAV5-EF1a-DIO-RFP into the EW region of Nmu-Cre mice and the protocol followed to prepare samples for imaging. RFP fluorescent signal was amplified using an anti-mCherry primary antibody and a Cy3-conjugated secondary antibody. The amplified RFP signal is shown in red in coronal slices and **C** in a sagittal view. Representative images from 1 mouse brain; n = 3 brains were analyzed. Images were acquired with a confocal microscope with a 10 × objective lens. Numbers denote neuroanatomical coordinates relative to Bregma. Scale bar coronal view: 300 µm. Scale bar sagittal view: 1000 µm. EW: Edinger–Westphal nucleus (PrEW) pre-nucleus, RLi: rostral linear nucleus of the raphe, VTA: ventral tegmental area, PAG: periaqueductal grey matter (LPAG) lateral part, Aq: aqueduct, PH: posterior hypothalamus, RFP: red fluorescent protein

In order to examine the expression of NMU-containing cells in the EW, we first analyzed brain sections from Nmu-Cre:ZsGreen1 mice. Coronal slices from -2.80 mm to -4.04 mm relative to Bregma showed ZsGreen1 cells from the midbrain region comprising the caudal portion of the posterior hypothalamus (PH) to the caudal EW. Green cells were also located dorsally, surrounding the aqueduct, mainly in the lateral periaqueductal gray matter (LPAG), and ventrally, in the anterior portion of the rostral linear nucleus of the raphe (RLi) and the parabrachial pigmented nucleus (PBP) of the ventral tegmental area (VTA) (Fig. 1A).

To further confirm the expression of NMU-containing cells in the abovementioned regions in adult mice and to exclude potential artefacts of the ZsGreen1 reporter, we performed intracerebral injections of the double-floxed AAV5-EF1a-DIO-RFP vector into the region comprised between the PH and the pre-Edinger–Westphal (PrEW) nucleus of Nmu-Cre mice. The analysis of coronal sections showed RFP-marked cells consistently located in the midbrain region comprising the caudal PH, EW, RLi and dorsal part of the PBP (Fig. 1B). Sagittal brain sections suggested a continuum of NMU-containing cells in the so-called subaqueductal paramedian zone [17] (Fig. 1C).

### NMU-Containing Cells in the Subaqueductal Paramedian Zone send Projections to the Frontal Part of the Dorsal Raphe Nucleus

**Fig. 2:**
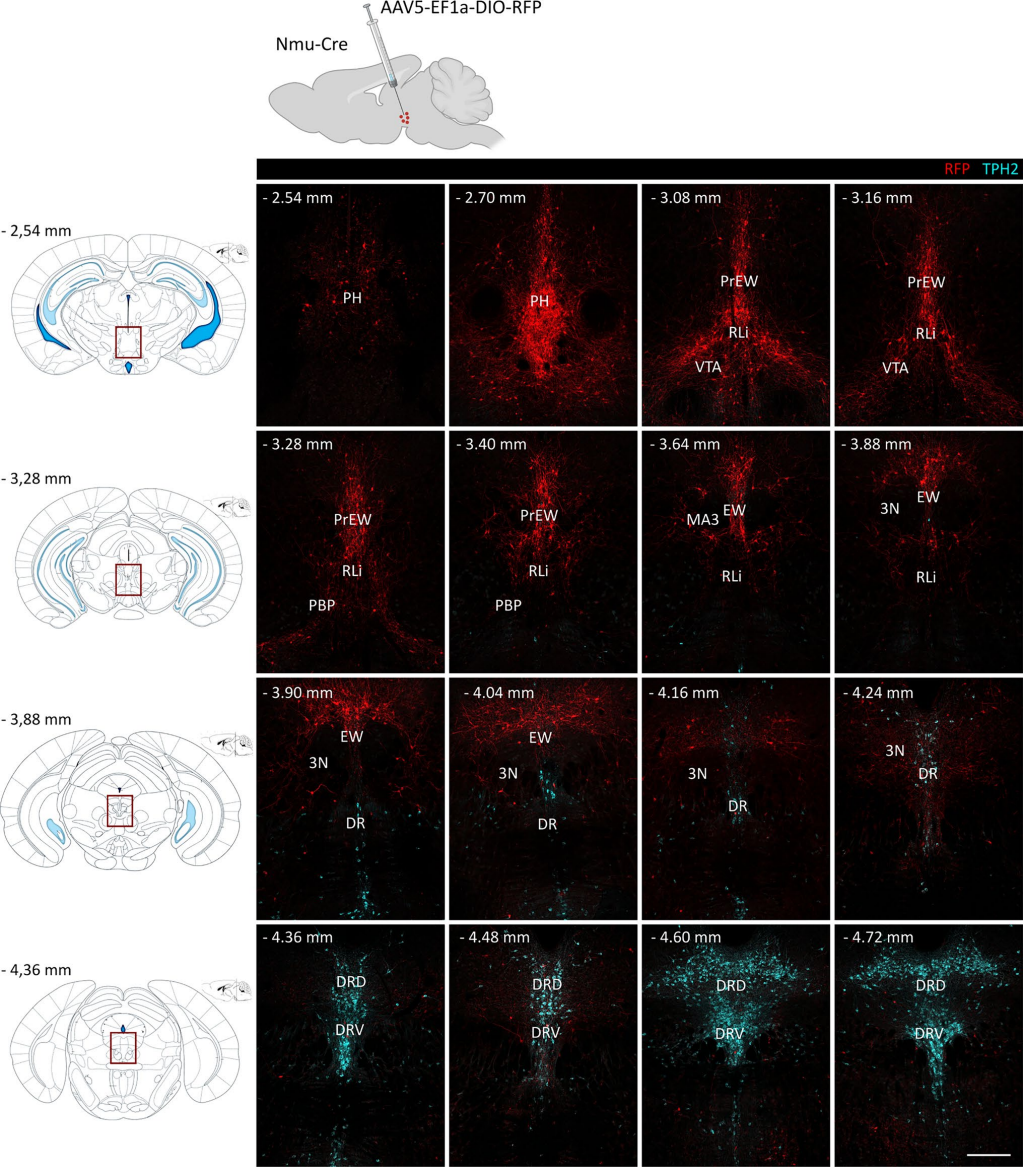
Distribution of NMU-producing cells in the region comprising the PH, EW and DR. Schematic representation of the intracerebral injection of the viral tracer AAV5-EF1a-DIO-RFP into the EW region of Nmu-Cre mice (top). Schematic representations of coronal slices with the region of interest outlined in red (left). RFP fluorescent signal (red) was amplified using an anti-mCherry primary antibody and a Cy3-conjugated secondary antibody. An antibody against TPH2 was used to mark serotoninergic neurons (light blue). Representative images from 1 mouse brain; *n* = 3 brains were analyzed. Images were acquired with a confocal microscope with a 10 × objective lens. Numbers denote neuroanatomical coordinates relative to Bregma. Scale bar: 300 µm. DAPI (not shown) was used to determine regional boundaries. PH: posterior hypothalamus, EW: Edinger–Westphal nucleus (PrEW) pre-nucleus, RLi: rostral linear nucleus of the raphe, VTA: ventral tegmental area, PBP: parabrachial pigmented nucleus of the VTA, MA3: medial accessory oculomotor nucleus, 3N: oculomotor nucleus, DR: dorsal raphe nucleus (DRD) dorsal part (DRV) ventral part, RFP: red fluorescent protein, TPH2: tryptophan hydroxylase 2

To study the extent of the NMU population, we selected brain sections of Nmu-Cre mice injected with AAV5-EF1a-DIO-RFP into the EW region. We took advantage of the reported anterograde transport ability of the AAV5 serotype [50–52] to study whether NMU-containing cells project to the DR nucleus, described as a site of action for NMU [53]. To do so, we examined serial coronal brain sections from − 2.54 mm to − 4.72 mm relative to Bregma using an anti-tryptophan hydroxylase 2 (TPH2) antibody as a marker of the serotonergic neurons in the DR, known to contain one of the largest groups of serotonin positive neurons in the brain. We observed a clear cluster of RFP cells in the PH (− 2.70 mm), moderate expression in but not restricted to the pre-EW and EW nucleus (− 3.08 to − 3.88 mm), extended ventrally to the dorsal part of the PBP (− 3.08 to − 3.28 mm) and surrounding the oculomotor nucleus (3N, − 3.40 to − 3.88 mm). Posterior levels showed RFP cells restricted ventrally to the aqueduct (− 3.90 to − 4.16 mm) and marked projections visible around the TPH2 immunoreactive cells, from − 4.04 to − 4.48 mm (Fig. 2). These results confirm that NMU-containing cells in the subaqueductal paramedian zone send projections to anterior parts of the DR nucleus.

We also examined the projection areas of the EWcp NMU cells in the forebrain. Contrary to the findings of Priest and co-workers [54] for EWcp CART neurons, we did not see RFP-positive nerve fibers in the nucleus accumbens, striatum and extended amygdala (images not shown).

### NMU Cells Minimally Co-localize with Other Markers in the EW and the VTA

Additional immunofluorescence analyses were performed to further characterize the continuum of NMU-containing cells in the subaqueductal paramedian zone.

At the neurochemical level, the EWcp can be identified by the expression of certain neuropeptides, such as CCK [21, 54], while cholinergic neurons, identified by choline acetyltransferase (ChAT) immunoreactivity, delineate the oculomotor nucleus. However, a few sparsely located ChAT + cells have also been described in the EW, possibly representing the EWpg [21]. The EWcp also contains a small population of tyrosine hydroxylase (TH) + cells [21].

**Fig. 3:**
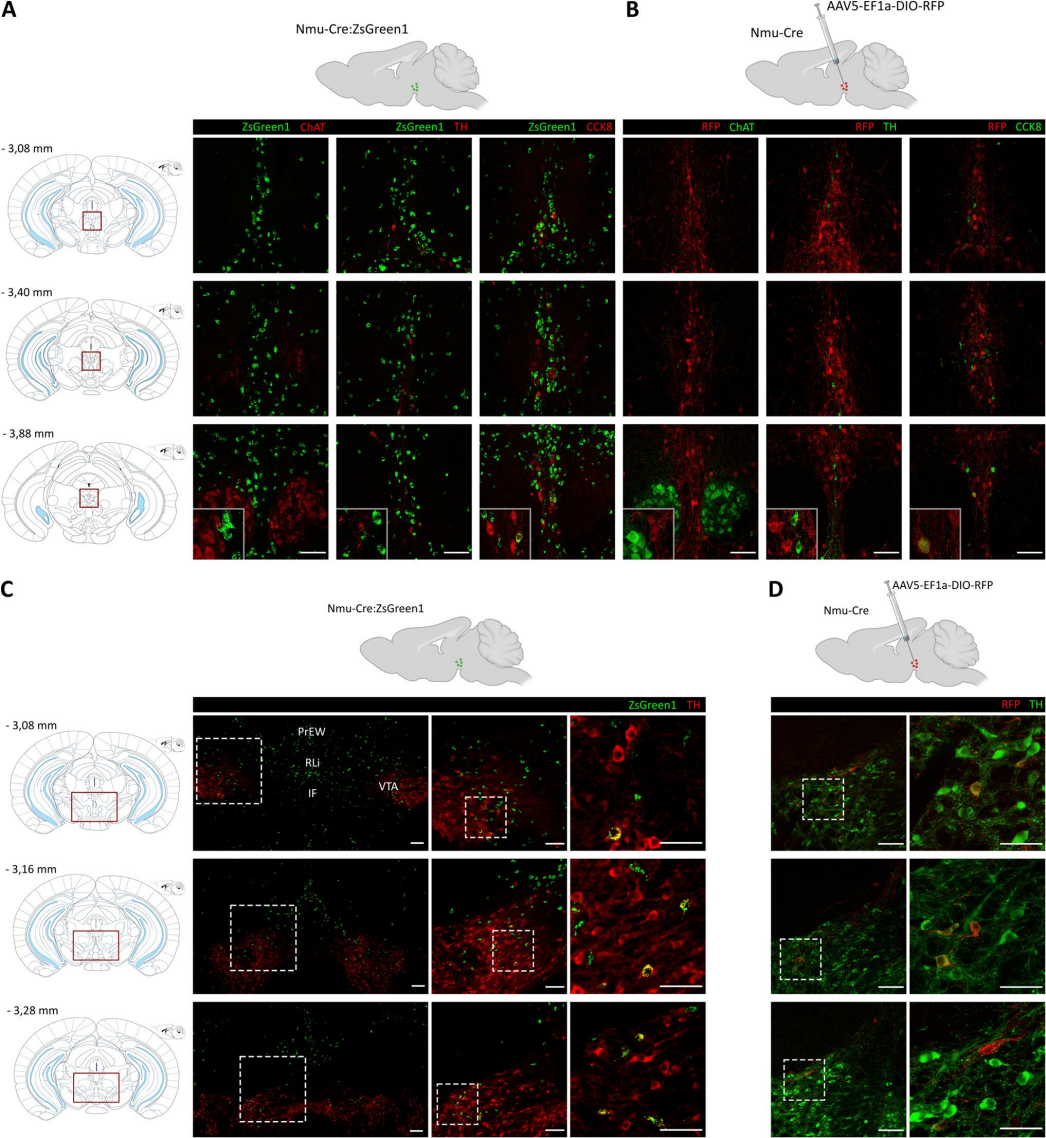
Characterization of NMU neurons in the EW and VTA using IHC. **A**–**D**, top: Schematic representation of a sagittal view of the brain of a Nmu-Cre:ZsGreen1 mouse and Nmu-Cre mouse injected with the viral tracer AAV5-EF1a-DIO-RFP into the EW region. **A**–**D** left: Schematic representations of coronal slices with the region of interest outlined in red. Numbers denote neuroanatomical coordinates relative to Bregma. **A**, **B** Expression of NMU-producing neurons in the region comprising the EW nucleus. **A** In green, endogenous ZsGreen1 fluorescence; in red, ChAT, TH and CCK8 (secondary antibody coupled to Cy5). **B** In red, RFP fluorescent signal amplified with an anti-mCherry primary antibody and a Cy3-conjugated secondary antibody; in green, ChAT, TH and CCK8 (secondary antibody coupled to Alexa Fluor 488). Representative images from 1 mouse brain; *n* = 3 brains were analyzed. Images were acquired with a confocal microscope with a 20× objective lens. Areas of higher magnification (40×) are outlined in grey (left-bottom). **C**, **D** Expression of NMU-producing neurons in the dorsal VTA. **C** In green, endogenous ZsGreen1 fluorescence; in red, TH (secondary antibody coupled to Cy5). The enlarged images (from left to right) are enclosed in a dashed box. Scale bar: 100 µm (left, middle) and 50 µm (right). (D) In red, RFP fluorescent signal amplified with an anti-mCherry primary antibody and a Cy3-conjugated secondary antibody; in green, TH (secondary antibody coupled to Alexa Fluor 488. Images were acquired with a confocal microscope with a 20× objective lens. The enlarged images (from left to right) are enclosed in a dashed box. Scale bar: 100 µm (left) and 50 µm (right). DAPI (not shown) was used to determine regional boundaries. PrEW: Pre-Edinger–Westphal nucleus, RLi: rostral linear nucleus of the raphe, IF: interfascicular nucleus, VTA: ventral tegmental area, RFP: red fluorescent protein, TH: tyrosine hydroxylase, CCK8: cholecystokinin-8, ChAT: choline acetyltransferase

We used antibodies against ChAT, CCK8 (a bioactive CCK fragment) and TH in coronal slices from Nmu-Cre:ZsGreen1 mice and Nmu-Cre mice injected with AAV5-EF1a-DIO-RFP into the EW region. The analysis of sections from − 3.08 to − 3.88 mm relative to Bregma revealed no overlap between ZsGreen1 and ChAT nor between ZsGreen1 and TH. Conversely, we observed limited overlap with CCK8. We found that 6,38% ± 2,21% (SEM) of CCK8 positive cells also contained ZsGreen1. Comparable results were observed with RFP, showing that 5,93% ± 1,46% (SEM) of CCK8 positive cells also contained RFP, while no overlap was found between RFP and ChAT or TH (Fig. 3A, B). These results suggested that NMU neurons constitute a distinct population from CCK + neurons and confirmed that NMU neurons are located in the midbrain region comprising the EW nucleus and delineated by the oculomotor nucleus, not restricted to the defined EWcp.

Since the NMU population extends ventrally to the dorsal part of the PBP, we used TH as a marker of dopaminergic cells to additionally examine the potential overlap with NMU in this region. The results showed scattered ZsGreen1-marked cells in the dorsomedial portion of the VTA (− 3.08 to − 3.16 mm) and the PBP (− 3.28 m), moderately overlapping with TH (Fig. 3D). Consistent results were obtained in brain sections from Nmu-Cre mice (Fig. 3D). These results confirmed the expression of NMU neurons in the VTA and suggested that a subpopulation of these neurons is dopaminergic.

### *Nmu* mRNA is Expressed in GABA- and Glutamatergic Neurons and Minimally Overlap with Other Peptidergic Neurons in the EW

**Fig. 4:**
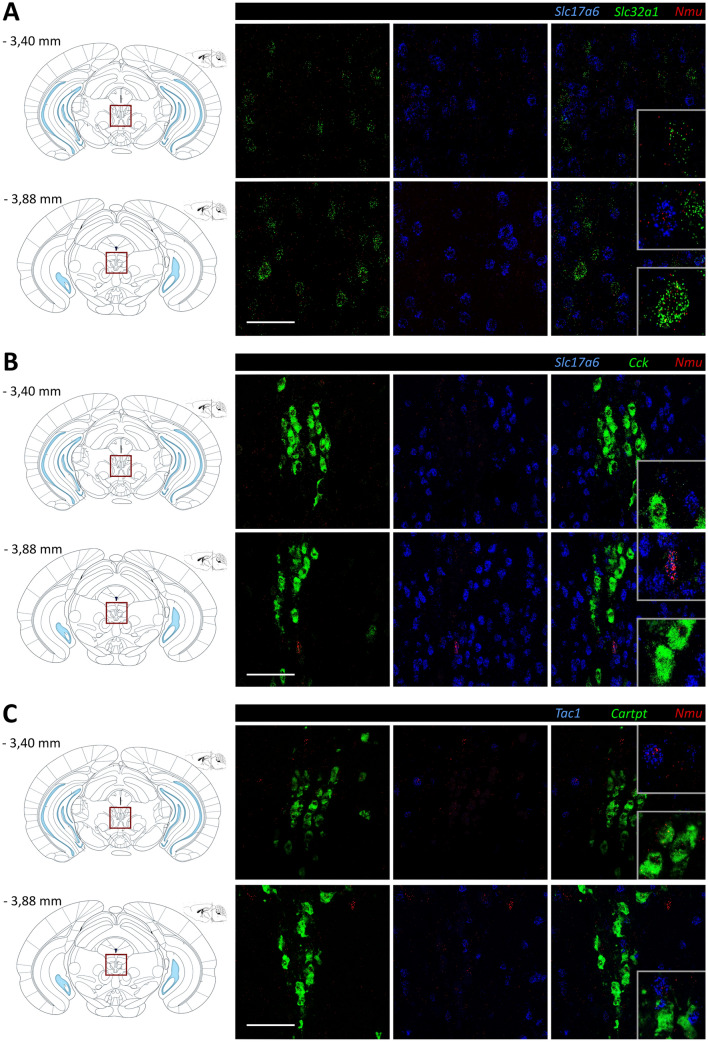
Characterization of NMU neurons in the EW using single-molecule fluorescent in situ hybridization (RNAscope®). **A** RNAscope® for *Slc17a6* (blue, VGLU2), *Slc32a1* (green, VIAAT) and *Nmu* (red) mRNA. **B** RNAscope® for *Slc17a6* (blue, VGLU2), *Cck* (green) and *Nmu* (red) mRNA. **C** RNAscope® for *Tac1* (blue), *Cartpt* (green) and *Nmu* (red) mRNA. Schematic representations of coronal slices with the region of interest outlined in red (**A**–**C**, left). Representative images from 1 mouse brain; *n* = 2 brains were analyzed. Images were acquired with a confocal microscope with a 40× objective lens. Areas of higher magnification are outlined in grey (right-bottom). Numbers denote neuroanatomical coordinates relative to Bregma. Scale bar: 50 µm. DAPI (not shown) was used to determine regional boundaries

The existence of obligatory peptidergic neurons in the EWcp has been suggested [54]. In this context, we addressed co-expression of the *Nmu* transcript with vesicular transporters of the primary excitatory and inhibitory neurotransmitters of the CNS, gamma-aminobutyric acid (GABA) and glutamate. Using single-molecule fluorescence in situ hybridization (ISH) by RNAscope®, we tested the presence of *Nmu*, *Slc17a6* (vesicular glutamate transporter VGLU2) and *Slc32a1* (vesicular inhibitory amino acid transporter VIAAT) in coronal brain slices from Nmu-Cre mice at the level of the EW (Fig. 4A). Our results showed *Nmu* mRNA in the soma but not restricted to it, possibly in axons and/or dendrites, as previously described in other brain regions [43]. The *Nmu* + cells analyzed appear to co-express the transcript for glutamate or GABA-glycine transporters, however, the presence of *Nmu*+cells expressing neither VGLU2 nor VIAAT cannot be ruled out.

We then examined the co-expression of *Nmu* mRNA with peptidergic transcripts previously described in the EW nucleus, such as *Cck*, *Tac1* and *Cartpt*. Examination of target mRNA in intact cells revealed limited overlap between *Nmu* and the transcripts analyzed, as well as *Nmu*+cells that apparently express neither *Cck*, nor *Tac1* or *Cartpt* (Fig. 4B, C). Taken together, our results confirmed the expression of *Nmu* mRNA in the EW region, even though the expression level was low except for a few cells with higher transcript content. Moreover, *Nmu* was present in neurons expressing fast-acting neurotransmitters and in other neuropeptidergic cells already described in the EWcp.

### A Subset of UCN1-Containing Cells in the EWcp Expresses NMU and *Nmu* mRNA

**Fig. 5:**
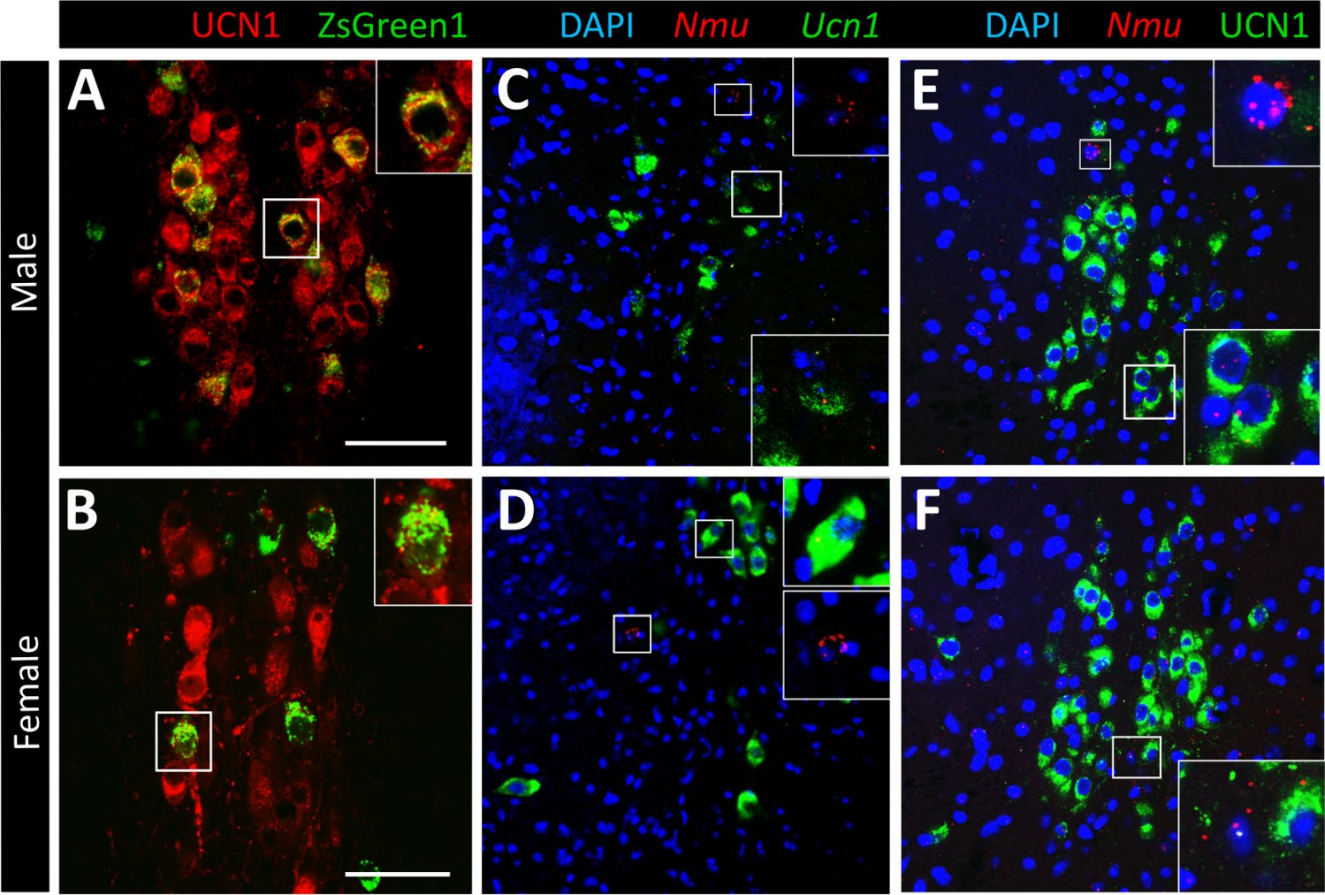
Assessment of *Ucn1* mRNA and UCN1 peptide content of NMU neurons in the EWcp area by immunofluorescence and RNAscope® in situ hybridization. Immunofluorescence for UCN1 peptide (red in **A** and **B**) in the EWcp of male (**A**) and female (**B**) Nmu-Cre:ZsGreen1 mice revealed that a subpopulation of UCN1-immunoreactive cells contained ZsGreen1 also in both sexes. RNAscope® for *Ucn1* mRNA (green in **C** and **D**) and *Nmu* mRNA (red in **C** and **D**) in male (**C**) and female (**D**) wild type mice confirmed the partial co-existence of these at the mRNA level. We saw also some *Nmu*-expressing cells that were negative for *Ucn1 *(*see insets*). In line with this, immunofluorescence for the UCN1 peptide (green in **E** and **F**) combined with RNAscope® for *Nmu* mRNA (red in **E** and **F**) revealed that the *Nmu* mRNA might be present, but it is not restricted to UCN1-containing neurons of the male and female EWcp. Nuclear counterstaining (blue in **C**–**F**) was performed with DAPI. Scale bars: 50 µm

Besides the addressed neuropeptides, the EWcp is the primary expression site of UCN1 [27, 28]. The functions of UCN1, such as reward processing, stress coping, energy homeostasis and regulation of feeding behavior, are comparable to those described for NMU, which also shares some expression sites of UCN1 and interacts with the CRH system [33–35, 43]. Based on this, the question arose whether EW peptidergic cells co-express NMU. To address this, we examined the UCN1 immunoreactivity in Nmu-Cre:ZsGreen1 mice, revealing that approximately 25% of the UCN1 immunoreactive cells also contain NMU. We did not observe any sex differences in the ratio of ZsGreen1 and UCN1 co-expressing neurons (Fig. 5A, B). Using RNAscope® ISH for *Nmu* and *Ucn1* mRNA in slices from wild type mice we observed a subset of urocortinergic cells containing *Nmu* mRNA (Fig. 5C, D). The quantification showed that 24,84% ± 1,20% of the *Ucn1* mRNA positive cells also contained *Nmu* mRNA. These results were confirmed when we performed the *Nmu* RNAscope® in combination with immunolabeling for UCN1 peptide (Fig. 5E, F). Overall, our results showed low expression of *Nmu* mRNA in the UCN1 neurons, however some cells in the EWcp area with higher *Nmu* mRNA content were identified as negative for both *Ucn1* mRNA and UCN1 peptide (Fig. 5C–F).

### Acute Alcohol Exposure Induces FOS Expression in a Subpopulation of NMU Neurons in the EW

**Fig. 6:**
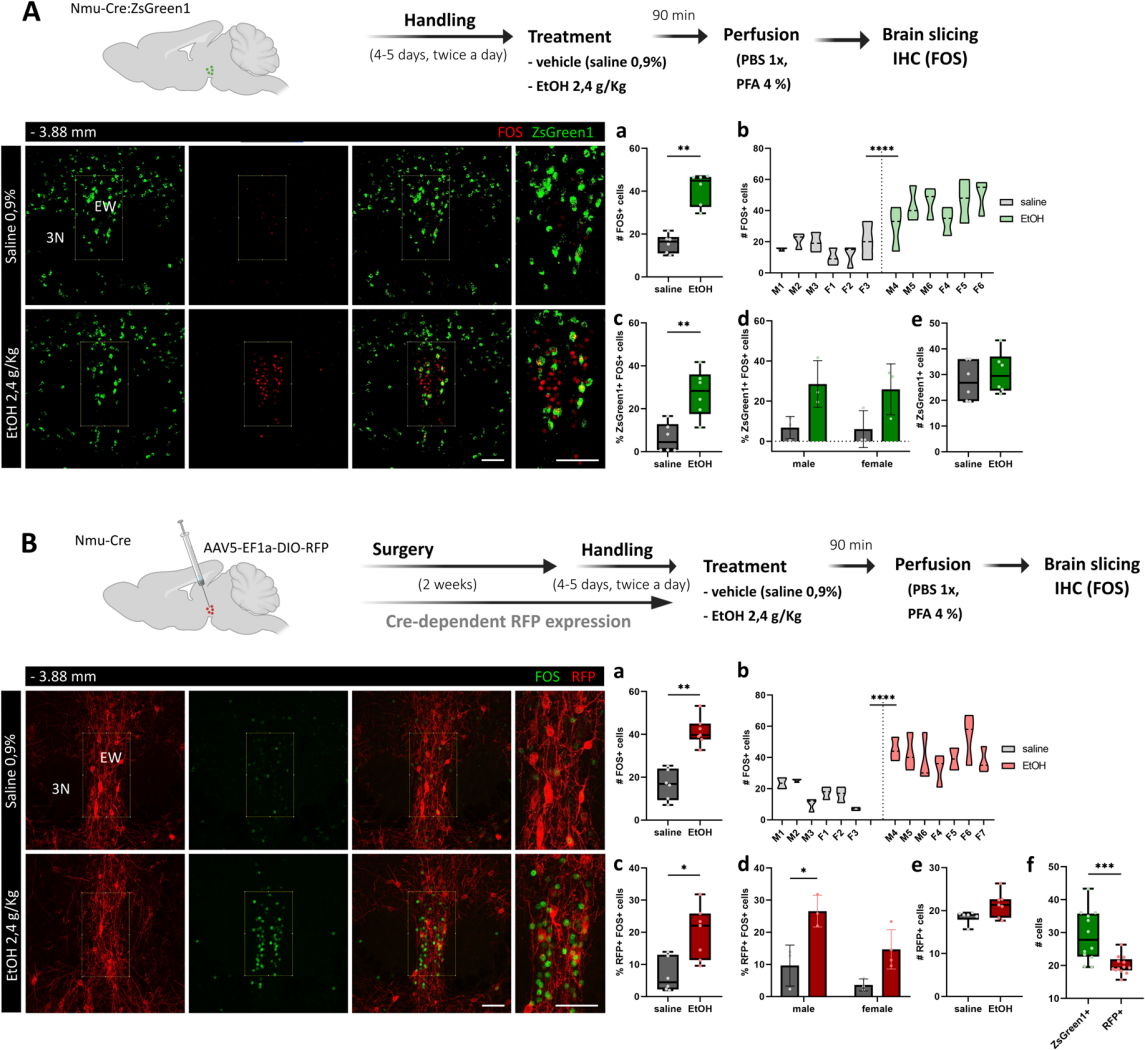
Analysis of FOS expression after acute alcohol exposure in NMU neurons in the EW region. **A** Schematic representation of the protocol followed to prepare samples from Nmu-Cre:ZsGreen1 for imaging. Coronal slices showing endogenous ZsGreen1 expression in green and FOS in red (secondary antibody coupled to Cy5). **A**-a Comparison of the FOS+cell count upon vehicle (0,9% NaCl) or ethanol (2,4 mg/kg) injection. Mann Whitney test, U = 0, **p = 0.0022. **A**-b Nested graph showing rostral, midline and caudal EW FOS+counts as technical replicates per mouse, considering male (M1-6) and female (F1-6) mice separately (nested t test, F = 43.93, ****p < 0.0001). **A**-c Percentage of ZsGreen1 and FOS co-expressing cells upon vehicle or ethanol injection (Mann–Whitney test, U = 2, **p = 0.0087). **A**-d The percentage of ZsGreen1 and FOS double-positive cells was influenced by the effect alcohol treatment (F_1,8_ = 12.64, p = 0.0075). **A**-e No differences between groups were observed in the amount of ZsGreen1 + cells (Mann Whitney test, U = 14, p = 0.5563). **B** Schematic representation of the intracerebral injection of the viral tracer AAV5-EF1a-DIO-RFP into the EW region of Nmu-Cre mice and the protocol followed to prepare samples for imaging. Coronal slices showing RFP fluorescent signal in red (amplified using an anti-mCherry primary antibody and a Cy3-conjugated secondary antibody) and FOS in green (secondary antibody coupled to Cy2). **B**-a Comparison of the FOS+cell count upon vehicle (0,9% NaCl) or ethanol (2,4 mg/kg) injection. Mann Whitney test, U = 0, **p = 0.0012. **B**-b Nested graph rostral, midline and caudal EW FOS + counts as technical replicates per mouse, considering male (M1-6) and female (F1-7) mice separately (nested t test, F = 40.98, ****p < .0001). **B**-c Percentage of RFP and FOS co-expressing cells upon vehicle or ethanol injection (Mann–Whitney test, U = 4, *p = 0.014). **B**-d The percentage of RFP and FOS double-positive cells was influenced by the effect of sex (F_1,9_ = 9.254, p = 0.014) and alcohol treatment (F_1,9_ = 22.65, p = 0.001). **B**-e No differences between groups were observed in the amount of RFP + cells (Mann Whitney test, U = 10, p = 0.1265). **B**-f Total number of RFP and ZsGreen1 cells confirmed the widespread patter of ZsGreen1 at the level of the EW (Mann–Whitney test, p = 0.0003). EW: Edinger–Westphal nucleus, 3N: oculomotor nucleus, RFP: red fluorescent protein. (A, B) Representative images from 1 mouse brain; *n* = 3–4 brains per experimental group and 3–4 slices per mouse were analyzed. Images were acquired with a confocal microscope with a 20× objective lens. The region of interest is outlined in white. Numbers denote neuroanatomical coordinates relative to Bregma. Scale bar: 100 µm. DAPI (not shown) was used to determine regional boundaries

The EWcp is known to be highly sensitive to alcohol administration and consumption [4, 11, 15, 16, 29]. Recently, a role of striatal NMU signaling in reward and alcohol consumption has been suggested [41]. However, whether NMU neurons are activated by acute alcohol exposure remains unknown. To address this question, we treated Nmu-Cre:ZsGreen1 male and female mice with a single i.p. injection of either vehicle or ethanol. After 90 min, the time required to achieve peak of FOS protein expression, mice were sacrificed, and their brains dissected for further analysis (Fig. 6A). Since no sex differences were observed in any of the analyses, male and female mice were grouped together. Quantification of FOS immunoreactivity confirmed that acute ethanol exposure induces FOS expression in the EWcp (Mann Whitney test, U = 0, p = 0.0022). Interestingly, the percentage of ZsGreen1+cells that co-express FOS was significantly increased in the alcohol group (Mann Whitney test, U = 2, p = 0.0087) while, considering male and female mice separately, the results showed a treatment effect (two-way ANOVA, F_1,8_ = 12.64, p = 0.0075) while the effect of sex did not reach statistical significance (two-way ANOVA, F_1,8_ = 0.08622, p = 0.07765). No difference was found in the total number of ZsGreen1+cells (Fig. 6A a–e).

Since reporter expression can be more widespread than anticipated [55, 56], as it has been described for Nmu-Cre:ZsGreen1 [43], we also examined the effects of acute alcohol administration in Nmu-Cre mice. Therefore, we injected male and female mice with AAV5-EF1a-DIO-RFP into the EW region and, after 3 weeks, they received a single i.p. injection of either vehicle or ethanol. The analysis of coronal brain sections at the level of the EW showed comparable results to that obtained in Nmu-Cre:ZsGreen1 mice (Fig. 6B). Acute ethanol exposure induced FOS expression in the EWcp (Mann–Whitney test, U = 0, p = 0.0012) and the percentage of RFP+cells that co-expressed FOS was significantly higher in the alcohol group (Mann–Whitney test, U = 4, p = 0.014). Interestingly, considering male and female mice separately, the analysis of the percentage of RFP and FOS co-expressing cells revealed a treatment effect (two-way ANOVA, F_1,9_ = 22.65, p = 0.001) but also a sex-effect (two-way ANOVA, F_1,9_ = 9.254, p = 0.014). Indeed, alcohol increased the cell count significantly in males but not in females (Tukey’s multiple comparisons test, p = 0.0149) (Fig. 6B a–e). Moreover, total number of RFP+cells was not different between groups but confirmed the widespread pattern of ZsGreen1 at this level (Mann–Whitney test, u = 16, p = 0.0003) (Fig. 6B f).

Taken together, these results showed that alcohol exposure induces FOS expression in the EWcp and suggested that around 10% of NMU neurons in the region comprising the EW are activated after a single ethanol injection.

### A Small Percentage of Alcohol-Induced FOS Cells in the EWcp Co-express UCN1 and NMU

Among the different neuropeptides expressed in the EWcp, previous results in rodents showed a primarily role of UCN1 in the control of ethanol intake and preference. Remarkably, alcohol-induced expression of FOS in the EWcp largely co-localize with UCN1 [4, 5, 29]. Notably, UCN1 levels in the rat EWcp have been shown to differ between males and females [57]. Here we showed that a subpopulation of UCN1 neurons expresses NMU. The question arises whether these cells respond to alcohol administration, and whether they do it in a possibly sex-dependent manner.

**Fig. 7:**
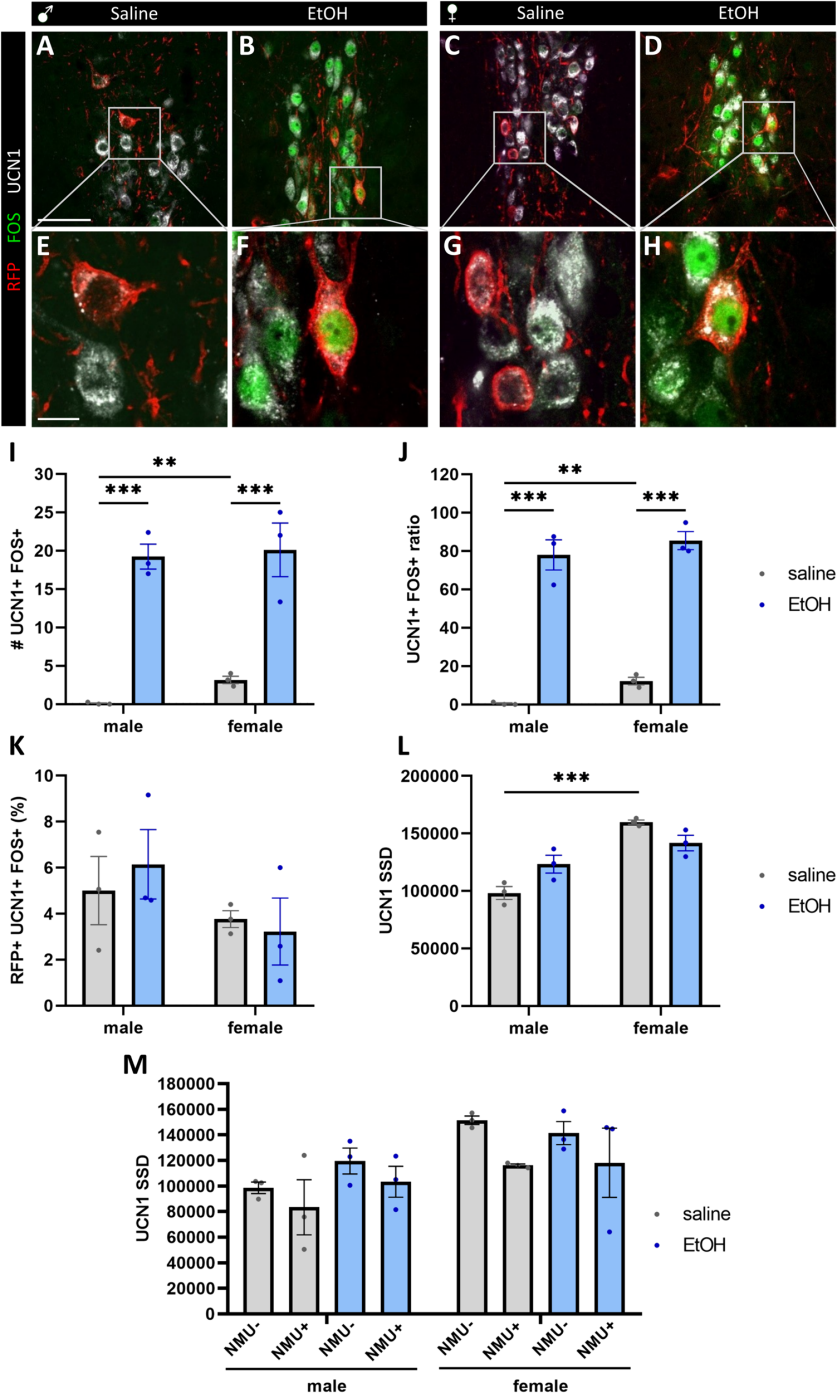
Analysis of UCN1 and FOS content of NMU cells upon alcohol treatment in the EW. Confocal images of triple immunofluorescence preparations **A**–**H** illustrate the nuclear FOS immunoreactivity (green) in UCN1-immunopositive (white) neurons that were sometimes positive for red fluorescent protein (RFP, red) referring to AAV5-EF1a-DIO-RFP virus infection upon EW injection in male and female Nmu-Cre mice. Panels **E**–**H** are higher magnification images of areas boxed in panels **A**–**D**. Histogram **I** illustrates the number of UCN1 and FOS positive cells in the EWcp. The ratio of UCN1 cells that were also positive for FOS is shown in panel **J**. Panel **K** illustrates that the ratio of RFP-expressing UCN1 neurons was not affected by sex or alcohol treatment. The effect of sex influenced the UCN1 specific signal density (SSD) in the EWcp is shown in **L**. Histogram **M** illustrates the UCN1 SSD in cells that contained NMU (RFP positive) or were negative for NMU (RFP negative). Scale bars: 50 µm in **A**–**D**, 10 µm in **E**–**H**

Coronal brain slices from Nmu-Cre mice injected earlier with AAV5-EF1a-DIO-RFP into the EW region were analyzed (Figure A-H). First, the count of UCN1 and FOS co-expressing cells confirmed that urocortinergic EWcp cells respond to alcohol (Fig. 7I). Interestingly, it was influenced by the main effect of sex (F_1,8_ = 11.33, p = 0.009) and alcohol treatment (F_1,8_ = 192.95, p = 10^–7^). In control animals, female mice showed a higher basal UCN1 and FOS positive cell count in the EWcp (p = 0.008). Alcohol increased the cell count significantly both in males (p < 0.001) and females (p < 0.001) and the sex difference disappeared upon alcohol exposure (Fig. 7I, p = 0.99). When we assessed the ratio of urocortinergic cells that were also FOS positive, we saw that most of the UCN1-neurons contained FOS upon alcohol treatment (Fig. 7J). The ratio was influenced by the main effect of sex (F_1,8_ = 25.70, p < 0.001) and alcohol treatment (F_1,8_ = 414.39, p < 10^–6^). In control animals, female mice showed a higher basal FOS immunoreactive UCN1 cell ratio in the EWcp (p = 0.001). Alcohol significantly increased the cell count in both males (p < 0.001) and females (p < 0.001) and the sex difference was abolished by alcohol (p = 0.81) again. The triple labeling for RFP, UCN1 and FOS revealed that 3–6% of the EWcp UCN1 neurons contained RFP also (Fig. 7K). The ratio of RFP and UCN1 co-expressing neurons was not influenced by the main effect of sex (F_1,8_ = 2.57, p = 0.14) or by alcohol treatment (F_1,8_ = 0.053, p = 0.82). Alcohol treatment showed no significant change in the proportion of RFP and UCN1 positive neurons in males (p = 0.92) or females (p = 0.99) and the sex difference by alcohol was insignificant (p = 0.43).

Taken together, our results confirmed that UCN1 neurons in the EWcp respond to alcohol exposure and reveal that a small subpopulation of UCN1+cells that also express NMU are sensitive to alcohol by means of FOS expression. Moreover, the ratio of RFP and UCN1 co-expressing neurons was not influenced by the sex of the animals or the treatment.

### UCN1 Peptide Signal Density Suggested that the Cells’ Peptide Content may be Affected by Alcohol in a Sex-Dependent Manner

In order to evaluate the cells’ UCN1 peptide content, we assessed the intensity of UCN1 immunofluorescence by SSD measurements in vehicle and alcohol-treated male and female Nmu-Cre mice upon EW injection of AAV5-EF1a-DIO-RFP. The assessment of UCN1 SSD in the EWcp (Fig. 7L) revealed the main effect of sex (F_1,8_ = 45.25, p = 0.0001) but not the alcohol treatment (F_1,8_ = 0.34, p = 0.57). Importantly, there was a strong interaction between the factors (F_1,8_ = 13.26, p = 0.006). Control female mice showed a significantly higher amount of UCN1 in the EWcp (p = 0.0005).

Next, we tested whether the NMU co-expressing RFP (NMU+) UCN1 cells differ in their UCN1 content from those cells that were not virus infected (NMU−). Our multifactorial ANOVA assessment revealed again that the UCN1 SSD in the cells was influenced by the sex (F_1,16_ = 45.25, p = 0.0001). It appeared that the UCN1 SSD was also affected by the NMU positivity (RFP immunoreactivity) of cells (F_1,16_ = 5.17, p = 0.037), while the main effect of alcohol on UCN1 SSD was not significant (F_1,16_ = 0.69, p = 0.41), and we also found no interactions between sex, treatment and NMU content (sex × treatment: F_1,16_ = 1.52; p = 0.23; sex × NMU content: F_1,16_ = 0.46; p = 0.50; treatment × NMU content: F_1,16_ = 0.07; p = 0.78; sex × treatment × NMU content: F_1,16_ = 0.10; p = 0.74). Despite the positive main effects, we did not see any significant differences in Tukey’s post hoc comparisons (Fig. 7M).

## Discussion

In the current study, we identified a continuum of NMU-producing cells in the subaqueductal paramedian zone, a midbrain region recently named by Pomrenze and colleagues [17]. This region comprises the EW (both centrally projecting -cp- and preganglionic -pg- populations), the region located immediately ventral to the PAG and the DR. It is composed by heterogeneous clusters of cells molecularly defined by the expression of neuropeptides such as UCN1, CCK and CART, as well as canonical neurotransmitters. Our results largely overlap with those described for the so-called subaqueductal paramedian zone, showing NMU neurons from the caudal part of the PH (− 2.54 mm) to the rostral part of the DR (− 4.24 mm), extending ventrally to the dorsal PBP (− 3.08 to − 3.28 mm) and dorsally surrounding the oculomotor nucleus (3N, − 3.40 to − 3.88 mm). This region also comprises the pre-EW and EW nucleus (− 3.08 to − 3.88 mm) as described in the atlas from Paxinos (Paxinos and Franklin [46]).

Focusing on the EW, the EWcp was shown to project to the spinal cord and multiple subcortical regions, such as the PAG, caudate-putamen, bed nucleus of stria terminalis, DR or lateral septum [3, 54, 58–60]. Even though it was thought that the EW did not project to brain regions typically associated with the rewarding properties of alcohol and addictive drugs, such as the amygdala and the nucleus accumbens [61], a recent study shows anterograde projections from the EWcp to the ventral striatum and central nucleus of the amygdala [54]. Our findings suggest that the NMU-expressing subpopulation of peptidergic EWpc cells do not provide rostral projections towards the forebrain, in contrast to other CART-expressing, but NMU-negative EWcp neurons.

The EWcp was shown to receive inputs from motor cortex, midbrain reticular nucleus, vestibular nucleus, PAG or lateral hypothalamus, among others [54]. Moreover, inputs from DR, locus coeruleus and VTA have been described before [62, 63], but a recent paper describing CART+EWcp connectivity did not find them [54]. This connectivity pattern supports a role in stress responses, pain modulation, eating behavior, addiction, and alcohol preference. Anatomically and functionally, there is a link between DR and EW that suggests a role in stress regulation and addiction-related behaviors [17, 60]. Taking advantage of the anterograde transport ability of the viral vector used to target NMU neurons, we observed projections from the continuum of NMU cells in the subaqueductal paramedian zone to the DR, a region that expresses type 2 NMU receptors and has been functionally described as a site of action for NMU [53]. Our results at the level of expression and connectivity support the potential role of NMU in addiction-related behaviors.

A unique combination of peptides is found in the EW region. Among them, UCN1 neurons show an almost complete overlap with CART [3, 4, 21, 54]. Interestingly, although some evidence suggests a large overlap between UCN1/CART and CCK [54], the distribution and co-expression of those peptides in the same neurons remains a matter of debate [21, 64]. Defining the EWcp by the expression of UCN1, our results showed NMU neurons in, but not restricted to, the EWcp. Interestingly, we found only a partial overlap of NMU cells and UCN1 cells, suggesting that NMU neurons in this region mainly constitute a different subpopulation of cells. We also analyzed the possible co-expression of NMU with other neuropeptides already described in the EWcp. Neurochemically, apart from UCN1, CCK and CART, the EWcp can be identified by the expression of other stress-, reward-, and energy expenditure-related neuropeptides, including SP and PACAP [65]. The immunolabeling of CCK8 revealed a very limited overlap with NMU-producing cells in the EW region. We performed ISH analyses by RNAscope® to detect *Cck*, *Tac1* (the gene coding for SP), and *Cartpt* (the gene coding for CART). Our results confirmed the expression of the *Nmu* transcript in, but not restricted to the EWcp, even though the expression was limited in comparison with the dense clusters of the other neuropeptidergic cells described in the region [17]. *Nmu* mRNA was only partially overlapping with *Cck*, *Tac1* and *Cartpt*. Similar results were found when we examined the potential co-expression of *Nmu* with markers of fast-acting neurotransmitters, such as VGLU2 and VIAAT vesicular transporters. The existence of peptidergic neurons in the EWcp has recently been addressed. Indeed, Priest and colleagues [54] suggested that CART+EW neurons may be an obligate peptidergic population. This population of cells contains multiple neuropeptides including CART, UCN1 and CCK, and lacks markers for fast neurotransmission. Although previously suggested in hypothalamic regions such as the paraventricular hypothalamic nucleus (PVN) [66], the existence of neuronal populations expressing only slow-acting neuropeptides remains a matter of debate. Here we succinctly addressed this possibility for *Nmu*-expressing neurons, showing *Nmu* mRNA expression in glutamatergic and GABAergic neurons. However, the low content and expression pattern of the *Nmu* transcript prevents any definitive conclusions from being drawn on this issue.

Considering the expression of NMU in the EW region, the partial overlap with UCN1 neurons and previous results that link NMU with alcohol-related behaviors, we decided to test whether NMU neurons were activated by acute alcohol exposure. Our results showed that a small percentage of NMU neurons in the EW region, around 10%, are activated by alcohol. Only the percentage of NMU activated neurons (RFP+cells that also expressed FOS) revealed a treatment effect and showed a possible sex effect, suggesting a more pronounced effect of alcohol in males compared to females. Similar results were found when analyzing triple-labeled cells, that is UCN1, RFP and FOS co-expressing cells. It should be noted that, although the differences described above were statistically significant, the absolute number of RFP and FOS double positive cells and UCN1, RFP and FOS co-expressing cells were low. Although the group size was limited, having 6–7 mice per group (vehicle *vs* treatment), 3–4 male or female mice per condition, the results suggested a possible differential effect of alcohol on male and female, which should be considered and addressed in future research. In this line, *Ucn1* mRNA content and UCN1 expression levels in the EWcp have been shown to differ between male and female rats [57]. Sex difference in the alcohol responsivity and the contribution to this phenomenon of the EWcp area is also supported by earlier studies where UCN1 knockout female mice were shown to exhibit lower alcohol preference than males, and the locomotor suppressing effect of alcohol in UCN1 knockout female mice is greater than in males [11].

Because the UCN1 content of the EWcp is affected by the estrous cycle [57], a limitation of the present work is that we did not determine the phase of the estrous cycle in female mice at the time of the experiment. This could mask possible differences in the comparison of UCN1 and NMU neuronal activation.

As to the comparison of UCN1 peptide content in NMU-expressing and NMU negative cells, one has to consider that we used RFP as a marker of NMU-expressing cells. As we saw in the Nmu-Cre:ZsGreen1 mice that about 25% of the green cells were UCN1+, but the ratio of RFP+cells that were UCN1+ was lower (4–6%), we could have identified some cells as NMU (RFP) negative. This could be due to the efficiency of viral transduction, which may result in false negative results. Moreover, while we found a significant main effect of sex and NMU presence, the post hoc comparisons did not confirm this. Future experiments on a larger sample using RNAscope® for *Nmu* and *Ucn1* combined with semi-quantitative immunofluorescence for UCN1 will help to address this uncertainty.

In summary, our study deepens our understanding of the expression and functionality of the NMU system. We described the expression of NMU-producing neurons forming a continuum of cells in the so-called subaqueductal paramedian zone, comprising the EW region. We provided proof for the expression of NMU within a subpopulation of UCN1 neurons in the EWcp, showing that this partial overlap does not interfere with the responsivity of UCN1-containing cells to alcohol. Our results also showed that a small subpopulation of NMU cells within the EW region responds to acute alcohol exposure, suggesting a differential sex-effect. Furthermore, we demonstrated that the neuron’s UCN1 content responds to alcohol in a sexually dimorphic way.

## Supplementary Information

Below is the link to the electronic supplementary material.

Supplementary file1 (TIF 5912 KB)—Suppl. Figure 1. Positive and negative controls for in situ hybridization (RNAscope®) labelling. Representative images of randomly selected sections of the EWcp area hybridized with triplex positive control probes for low- (DNA-directed RNA polymerase II subunit RPB1 mRNA, *Polr2a*), mid- (Peptidyl-prolyl cis–trans isomerase B mRNA, *Ppib*) and high-copy (Polyubiquitin-C mRNA, *Ubc*) (upper panel). Representative images of randomly selected sections of the EWcp area hybridized with a negative control probe against the bacterial dihydrodipicolinate reductase (*dabP*) mRNA (lower panel). Nuclear counterstaining (blue) was performed with DAPI. Scale bars: 50 µm

## Data Availability

No datasets were generated or analysed during the current study.

## Abbreviations

AAV: Adeno-associated virus
3N: Oculomotor nucleus
ANOVA: Analysis of variance
CART: Cocaine- and amphetamine-regulated transcript
CCK: Cholecystokinin
ChAT: Choline acetyltransferase
CNS: Central nervous system
CRH: Corticotropin-releasing hormone
DAPI: 4’,6-Diamidino-2-phenylindole dihydrochloride
DR: Dorsal raphe nucleus
DRD: Dorsal raphe nucleus, dorsal part
DRV: Dorsal raphe nucleus, ventral part
EW: Edinger–Westphal nucleus
EWcp: Edinger–Westphal nucleus, centrally projecting
EWpg: Edinger–Westphal nucleus, preganglionic
GABA: Gamma-aminobutyric acid
IHC: Immunohistochemistry
ISH: In situ hybridization
LPAG: Lateral periaqueductal gray matter
MA3: Medial accessory oculomotor nucleus
NMU: Neuromedin U
PACAP: Pituitary adenylate cyclase-activating polypeptide
PAG: Periaqueductal gray matter
PBP: Parabrachial pigmented nucleus of the VTA
PBS: Phosphate-buffered saline
PFA: Paraformaldehyde
PH: Posterior hypothalamus
PrEW: Pre-Edinger–Westphal nucleus
PVN: Paraventricular hypothalamic nucleus
RFP: Red fluorescent protein
RLi: Rostral linear nucleus of the raphe
SP: Substance P
SSD: Specific signal density
TB: Tris buffer
TBS: Tris-buffered saline
TBS-T: Tris-buffered saline-Triton-X
TH: Tyrosine hydroxylase
TPH2: Tryptophan hydroxylase 2
UCN1: Urocortin 1
VGLU2: Vesicular glutamate transporter 2
VIAAT: Vesicular inhibitory amino acid transporter
VTA: Ventral tegmental area
