# Supplementary figures and images for: Neuromedin U Neurons in the Edinger–Westphal Nucleus Respond to Alcohol Without Interfering with the Urocortin 1 Response
Source: Neurochem Res. 2024 Sep 12;49(12):3277–96. doi: 10.1007/s11064-024-04238-1 (PMC11502588; doi:10.1007/s11064-024-04238-1)

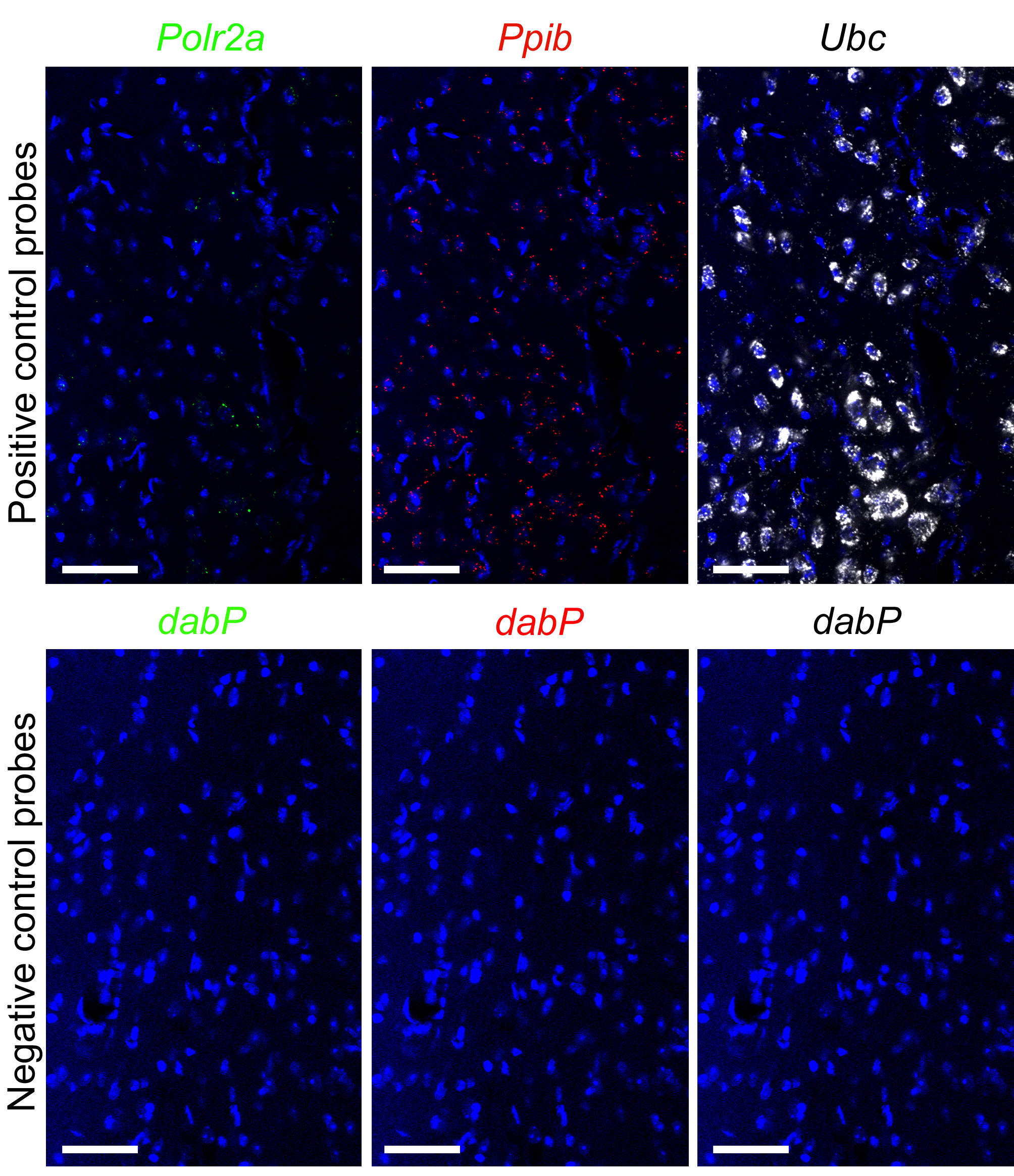

Supplement: Supplementary file 1 — Supplementary file1 (TIF 5912 KB)—Suppl. Figure 1. Positive and negative controls for in situ hybridization (RNAscope®) labelling. Representative images of randomly selected sections of the EWcp area hybridized with triplex positive control probes for low- (DNA-directed RNA polymerase II subunit RPB1 mRNA, Polr2a), mid- (Peptidyl-prolyl cis–trans isomerase B mRNA, Ppib) and high-copy (Polyubiquitin-C mRNA, Ubc) (upper panel). Representative images of randomly selected sections of the EWcp area hybridized with a negative control probe against the bacterial dihydrodipicolinate reductase (dabP) mRNA (lower panel). Nuclear counterstaining (blue) was performed with DAPI. Scale bars: 50 µm [file 11064_2024_4238_MOESM1_ESM.tif]
